# Chronic Genotype 3 Hepatitis E in Pregnant Woman Receiving Infliximab and Azathioprine

**DOI:** 10.3201/eid2405.171845

**Published:** 2018-05

**Authors:** Caroline Charre, Christophe Ramière, Jérôme Dumortier, Florence Abravanel, Sébastien Lhomme, Rodica Gincul, Caroline Scholtès

**Affiliations:** Hospices Civils de Lyon, Lyon, France (C. Charre, C. Ramière, J. Dumortier, R. Gincul, C. Scholtès);; Claude Bernard University Lyon 1, Villeurbanne, France (C. Charre, C. Ramière, J. Dumortier, C. Scholtès);; INSERM, Lyon (C. Charre, C. Ramière, C. Scholtès);; INSERM, Toulouse, France (F. Abravanel, S. Lhomme);; Centre Hospitalier Universitaire de Purpan, Toulouse (F. Abravanel, S. Lhomme);; Université Paul Sabatier, Toulouse (F. Abravanel, S. Lhomme)

**Keywords:** Hepatitis E virus, pregnancy, immunosuppression, viruses, chronic infection, hepatitis E

## Abstract

Acute hepatitis E virus infection during pregnancy has a high fatality rate in developing countries. Little data are available on chronic infection in pregnant women. We report a case of chronic hepatitis E during treatment with infliximab and azathioprine, without adverse event during pregnancy and with spontaneous resolution after delivery.

Hepatitis E virus (HEV) genotype 1 causes a high number of deaths of pregnant women in developing countries (*1*). The few reported cases of HEV during pregnancy in industrialized countries (*2*–*5*) mainly relate to acute genotype 3 infection. We report the course of autochthonous chronic genotype 3c (GenBank accession no. KX602217) hepatitis E in a pregnant woman in France.

The patient, 27 years of age, was receiving immunosuppressive therapies for ulcerative colitis and became pregnant during the infection and treatment. At symptom onset, she had received infliximab and azathioprine for >5 years and reported eating undercooked meat; she had not traveled abroad. Prolonged elevated alanine aminotransferase (ALT) since May 2014 led her physician to suspect viral hepatitis; HEV infection was later diagnosed in September 2014 by detection of HEV IgM and RNA in plasma (Figure, panel A). We retrospectively tested previous blood samples from this patient, routinely stored in the hospital virology laboratory, and found them to be negative for HEV IgM and RNA. 

**Figure:**
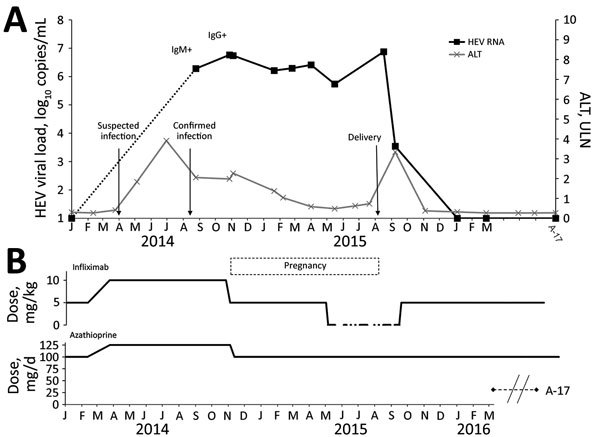
Time courses of HEV viral load (A) and infliximab and azathioprine treatment (B) in pregnant woman with chronic hepatitis E who was undergoing immunosuppressive treatment for ulcerative colitis. HEV RNA levels, IgM and IgG positivity, and serum ALT levels are shown. Serum ALT is expressed as a multiple of ULN. Arrows indicate suspected and confirmed infection and delivery dates. Infliximab treatment occurred every 8 weeks. ALT, alanine aminotransferase; HEV, hepatitis E virus; ULN, upper limit of normal.

Persistence of HEV has not been reported among patients receiving infliximab or azathioprine. However, HEV persistence was reported in a patient receiving azathioprine combined with oral steroids (*6*) and in a pig model of HEV chronicity under combined cyclosporine/azathioprine/methylprednisolone (*7*). On the basis of those reports, we reduced the patient’s dose of azathioprine to 100 mg/d and that of infliximab to 5 mg/kg/d every 8 weeks in November 2014 (Figure, panel B), but infection did not resolve. She became pregnant shortly thereafter. 

During the patient’s pregnancy, viral loads ranged from 5.7 to 6.8 log_10_ copies/mL, and ALT returned to reference range (Figure, panel A). We discontinued infliximab at the beginning of the third trimester (Figure, panel B). Viral load increased by >1 log_10_ copies/mL, and ALT remained within reference range. She gave birth by vaginal delivery at 40 weeks of amenorrhea. On the day of delivery, 3 months after infliximab discontinuation, viral load peaked (6.9 log_10_ copies/mL; Figure, panel A). Although viral load was high during pregnancy, the infant was not infected and was in good health; HEV RNA was undetectable in cord blood (the placenta was not available for evaluation), and neither HEV IgM nor RNA were found in the newborn’s plasma 2 days after birth. 

After delivery, testing of the mother’s plasma showed cytolysis (ALT >3× upper limit of reference range) and a >3-log decrease of HEV RNA (Figure, panel B). We reintroduced infliximab 3 weeks after delivery, at which time HEV RNA was lower than during pregnancy but still detectable (3.5 log_10_ copies/mL; Figure, panel A). At 2 months after delivery, hepatic cytolysis resolved; 2 months later, HEV became undetectable. No relapse was noted during subsequent follow-up (the last PCR performed in August 2017 was negative).

Because of the high rate of severe acute hepatitis E reported in pregnant women in developing countries, we monitored the patient for negative outcomes during gestation but found none. This finding is consistent with the small number of reported HEV infections during pregnancy in industrialized countries (*2*–*5*), despite high seroprevalence in the general population (*8*,*9*).

Innate immunity has been suggested as essential for severe outcomes of acute HEV infection during pregnancy (*10*). In our study, HEV infection had moved toward chronic infection before pregnancy, which may have reduced the role of innate immunity. T-cell responses are decreased in immunosuppressed patients and in pregnant women, particularly when term approaches. The imbalance in T-cell immunity (Th1/Th2) has been proposed to be implicated in the progression of chronic HEV infection in immunocompromised pigs (*7*). This imbalance may explain the absence of cytolysis during pregnancy and the increased viral load observed despite discontinuation of infliximab. Conversely, after delivery, restoration of cellular immunity is commonly observed (*11*) and may have contributed to efficient clearance of the virus by hepatic cytolysis along with the reduced immunosuppression resulting from infliximab discontinuation. Despite reintroduction of infliximab when HEV RNA was still detectable, we observed spontaneous resolution of chronic hepatitis E, although immunosuppressive treatment at that time was identical to that previously implicated in the chronicity of infection.

The risk for HEV vertical transmission seems dependent on viral load (*12*). In a model of HEV infection in pregnant rabbits, Xia et al. reported severe outcomes and a high level of transmission to offspring (*13*). In the case we report, despite high viral loads in the mother’s plasma throughout pregnancy, we found no HEV RNA in the newborn’s plasma. Of note, although mothers in the rabbit model were negative for HEV IgG throughout pregnancy, in the case we report, the mother was IgG positive before pregnancy, which may have helped protect the fetus from infection, although this protective role is inconsistent in previous reports of HEV genotype 3 (HEV3) infection of humans (*2*–*4*). Furthermore, despite a high sequence similarity to HEV3, rabbit HEV cross-species infections are restricted to nonhuman primates, and pathogenesis may differ from that of HEV3. In conclusion, our results and those reported by Mallet et al. (*5*) indicate that chronic HEV3 infection in pregnant women might resolve after pregnancy.
